# Long Non-Coding RNAs Modulate Sjögren’s Syndrome Associated Gene Expression and Are Involved in the Pathogenesis of the Disease

**DOI:** 10.3390/jcm8091349

**Published:** 2019-09-01

**Authors:** Marzia Dolcino, Elisa Tinazzi, Claudio Vitali, Nicoletta Del Papa, Antonio Puccetti, Claudio Lunardi

**Affiliations:** 1 Department of Medicine, University of Verona, Piazzale L.A. Scuro 10, 37134 Verona, Italy; 2 Sections of Rheumatology, Villa S. Giuseppe, Como and Casa di Cura di Lecco, 23900 Lecco, Italy; 3 U.O.C. Day Hospital Reumatologia, ASST G. Pini-CTO, 20122 Milan, Italy; 4 Department of Experimental Medicine, Section of Histology, University of Genova, Via G.B. Marsano 10, 16132 Genova, Italy

**Keywords:** primary Sjögren’s syndrome, long non-coding RNA, signaling pathway, protein–protein (PPI) network, gene module

## Abstract

Primary Sjögren’s syndrome (pSjS) is a chronic systemic autoimmune disorder, primarily affecting exocrine glands; its pathogenesis is still unclear. Long non-coding RNAs (lncRNAs) are thought to play a role in the pathogenesis of autoimmune diseases and a comprehensive analysis of lncRNAs expression in pSjS is still lacking. To this aim, the expression of more than 540,000 human transcripts, including those ascribed to more than 50,000 lncRNAs is profiled at the same time, in a cohort of 16 peripheral blood mononuclear cells PBMCs samples (eight pSjS and eight healthy subjects). A complex network analysis is carried out on the global set of molecular interactions among modulated genes and lncRNAs, leading to the identification of reliable lncRNA-miRNA-gene functional interactions. Taking this approach, a few lncRNAs are identified as targeting highly connected genes in the pSjS transcriptome, since they have a major impact on gene modulation in the disease. Such genes are involved in biological processes and molecular pathways crucial in the pathogenesis of pSjS, including immune response, B cell development and function, inflammation, apoptosis, type I and gamma interferon, epithelial cell adhesion and polarization. The identification of deregulated lncRNAs that modulate genes involved in the typical features of the disease provides insight in disease pathogenesis and opens avenues for the design of novel therapeutic strategies.

## 1. Introduction

Sjögren’s syndrome (SjS) is a chronic systemic autoimmune disease of still unknown origin, that may be present either alone (defined as primary SjS: pSjS) or associated with other autoimmune diseases (defined as secondary SjS). The prevalence of pSjS in the general population is between 0.01 and 0.1% with a higher prevalence of the disease in women [1,2]. The clinical symptoms of pSjS include exocrinopathy, resulting in dry eyes and mouth and extraglandular systemic manifestations such as arthralgias, fatigue, vasculitis, pulmonary fibrosis and pulmonary hypertension, interstitial nephritis and central and peripheral involvement of the nervous system [3].

Although little is known on the pathogenesis of the disease, several factors have been shown to contribute to its onset, such as genetic background, environmental factors including viral infections [4] and epigenetic mechanisms, such as microRNAs (miRNAs).

Our attention is focused on the epigenetic mechanisms that may be involved in pSjS pathogenesis, through the analysis of the modulation of long non-coding RNAs (lncRNAs) expression in pSjS patients.

The identification of lncRNAs modulated in the disease and an integrated analysis of lncRNAs, miRNAs and gene expression profiles in patients affected by pSjS are reported here as an historical first, to the best of the authors’ knowledge. Interestingly, the identified lncRNAs are able to modulate pathogenetically relevant molecular pathways of the disease.

## 2. Materials and Methods

### 2.1. Patients

Eight female patients (mean age 52 ± 15 years old) affected by pSjS, attending the Unit of Autoimmune Diseases at the University Hospital of Verona, Northern Italy, and 8 sex and age matched healthy controls were enrolled. Both patients and controls were subjects of Caucasian origin from Northern Italy. All patients enrolled in this study were diagnosed according to the American College of Rheumatology (ACR)–European League against Rheumatism (EULAR) criteria [5]. They underwent labial salivary gland biopsy and disease activity was evaluated at the moment of enrollment in the study.

A written informed consent was obtained from all the participants of the study and the study protocol was approved by the Ethical Committee of the Azienda Ospedaliera Universitaria Integrata di Verona (identification code 1538, version 3, date of approval 12 March 2012). All the investigations have been performed according to the principles contained in the Helsinki declaration.

### 2.2. Microarray Analysis

Blood sample collection was carried out using BD Vacutainer K2EDTA tubes (Becton Dickinson, Franklin Lakes, NJ, USA) and 21-gauge needles.

PBMCs isolation was performed by Ficoll–HyPaque (Pharmacia Biotech, Piscataway, NJ, USA) gradient centrifugation. Patients and controls had similar PBMCs distribution. Total RNA was extracted from PBMCs (10^7^ cells) using an miRNeasy mini kit (Qiagen GmbH, Hilden, Germany). cRNA preparation, samples hybridization and scanning were performed following the Affymetrix (Affymetrix, Santa Clara, CA, USA) provided protocols using a Cogentech Affymetrix microarray unit (Campus IFOM IEO, Milan, Italy). All samples were hybridized on a Human Clariom D (Thermo Fisher Scientific, Waltham, MA, USA) gene chip. Signal intensities were analysed with the Transcriptome Analysis Console (TAC) 4.0 software (Applied Biosystem, Foster City, CA, USA by Thermo Fisher Scientific, Waltham, MA, USA). 

Using the Human Clariom D arrays, more than 540,000 human transcripts were interrogated, starting from as little as 100 pg of total RNA. Signal intensity was background-adjusted, normalized, and log-transformed using the Signal Space Transformation (SST)-Robust Multi-Array Average algorithm (RMA). 

Differentially expressed genes that showed an expression level at least 1.5 fold different in the test sample versus control sample at a significant level (*p* ≤ 0.01) were chosen for final consideration. *p*-values were calculated applying the unpaired *t*-test and Bonferroni multiple testing correction.

Target annotations of long non-coding RNAs were retrieved using starBase v2.0 (http://starbase.sysu.edu.cn/) where lncRNAs interactions, experimentally validated by high-throughput experimental technologies, are registered [6].

The list of gene targets of microRNAs (miRNAs) that were targeted by lncRNAs were inferred interrogating the FunRich database (www.funrich.org/) [7].

### 2.3. Protein–Protein Interaction (PPI) Network Construction and Network Clustering

The PPI network was constructed upon the experimentally validated protein–protein interactions using STRING (Search Tool for the Retrieval of Interacting Genes) version 10.5 (http://string-db.org/) [8].

Network topological analysis was performed using the Cytoscape software (version 3.7, (www.cytoscape.org) [9].

High-flow areas (highly connected regions) of the network (modules) were detected using the MCODE plugin of Cytoscape (k-core = 4 and node score cutoff = 0.2).

### 2.4. Gene Functional Classification and Enrichment Analysis

Genes were classified functionally into Biological Processes (BPs) according to the Gene Ontology (GO) project annotations (www.geneontology.org) by the Panther expression analysis tools (http://pantherdb.org/) [10].

Pathway classification and enrichment (hypergeometric *p*-value ≤ 0.05) analysis were achieved with FunRich.

### 2.5. Real Time PCR of lncRNAs

Five hundred ng of total RNA was treated with 1 unit of DNase I Amplification Grade (Invitrogen; Carlsbad, CA, USA). First-strand cDNA was generated using the SuperScript IV First-Strand Synthesis System (Invitrogen; Carlsbad, CA, USA) with random hexamers, according to the manufacturer’s protocol. Real time PCR was performed in triplicate with PowerUp™ Sybr^®^ Green reagent (Applied Biosystems; Foster City, CA, USA) in a QuantStudio 6 Flex system (Applied Biosystems; Foster City, CA, USA). Transcripts of relative expression levels were obtained after normalization against the geometric mean of the housekeeping genes Glyceraldehyde 3-phosphate dehydrogenase GAPDH and beta-actin (ACTB) expression. The ΔΔCt method of relative quanrtification was used for comparing relative fold expression differences. Results are expressed as fold changes with respect to healthy subjects. 

### 2.6. Real Time PCR of Genes Modulated in pSjS Patients

First-strand cDNA was obtained using the SuperScript III First-Strand Synthesis System for RT-PCR Kit (Invitrogen), with random hexamers, following the manufacturer’s protocol. PCR was performed in a total volume of 25 μL containing 1× Taqman Universal PCR Master mix, no AmpErase UNG and 2.5 μL of cDNA; pre-designed, gene-specific primers and probe sets for each gene were obtained from Assay-on-Demande Gene Expression Products service (Applied Biosystems).

Real-Time PCR reactions were carried out in a two-tube system and in singleplex. The Real- Timeamplifications encompassed 10 min at 95 °C (AmpliTaq Gold activation), followed by 40 cycles at 95 °C for 15 s and at 60 °C for one minute. Thermocycling and signal detection were performed with a 7500 Sequence Detector (Applied Biosystems). Signals were detected following the manufacturer’s instructions. This methodology allowed the identification of the cycling point where the PCR product was detectable by means of fluorescence emission (Threshold cycle or Ct value). The Ct value correlated to the quantity of target mRNA. Relative expression levels were calculated for each sample after normalization against the housekeeping genes GAPDH, beta-actin and 18 s ribosomal RNA (rRNA) using the ΔΔCt method for comparing relative fold expression differences. Ct values for each reaction were determined using TaqMan SDS analysis software. Each amount of RNA tested had triplicate Ct values averaged. Since Ct values varied linearly with the logarithm of the amount of RNA, this average represented a geometric mean.

### 2.7. Real Time PCR of microRNAs

miRNAs expression was evaluated by TaqMan^®^ Advanced miRNA assays chemistry (Applied Biosystems, Foster City, CA, USA). Briefly, 10 ng of total RNA was reverse transcribed and pre-amplified with a TaqMan^®^ Advanced miRNA cDNA synthesis kit according to the manufacturer’s instructions (Applied Biosystems, Foster City, CA, USA). Pre-amplified cDNA was diluted 1/10 in nuclease-free water and 5 µL of diluted cDNA for each replicate was loaded in PCR. Twenty µL PCR reactions were composed by 2× Fast Advanced Master Mix and TaqMan^®^ Advanced miRNA assays for miR-30e-5p, miR-32-5p, miR-155-5p, miR-195-5p, miR-378a-3p and miR-30b-5p. The mean of Ct for hsa-miR-16-5p and hsa-miR-26a-5p expression was used to normalize miRNA expression. Real time PCR was carried out in triplicate on a QuantStudio 6 Flex instrument (Applied Biosystems, Foster City, CA, USA). Expression values were reported as fold change with respect to healthy controls by ΔΔCt method employing QuantStudio Real-Time PCR system software v. 1.3.

## 3. Results

### 3.1. Patients Characteristics

All the patients enrolled in the study had antinuclear antibodies >1:640, nuclear dots or homogeneous patterns, and were positive for anti-ENA antibodies SSA/Ro; four of them also were positive for anti-SSB antibodies. Labial salivary gland histopathology showed a focal lymphocytic sialadenitis with a focus score >1 [11]. The median Eular Sjogren’s syndrome disease activity index (ESSDAI) score was 6. The median Eular Sjogren’s syndrome Patient Reported Index (ESSPRI) score was 7 [12]. Three patients presented germinal centre-like structures in their labial salivary gland biopsy and one patient developed a B cell lymphoma soon after enrollment in the study.

### 3.2. High-Throughput Gene and Long Non-Coding RNA Expression Profiling in Peripheral Blood Mononuclear Cells of Patients with pSjS

Intending to identify lncRNAs potentially involved in pSjS pathogenesis, the expression of more than 540,000 human transcripts, including those ascribed to more than 50,000 lncRNAs, was profiled at the same time, in a cohort of 16 PBMCs samples (8 pSjS and 8 healthy subjects). Transcriptional profiles of pSjS patients and healthy subjects were compared and, after a robust filtering procedure (Bonferroni-corrected *p*-value ≤ 0.01 and fold change ≥ |1.5|), 2503 coding-genes were modulated significantly (Table S1).

The functional classification by Gene Ontology (http://www.geneontology.org/) of the 2503 differentially expressed genes (DEGs) revealed that they were involved in meaningful biological processes (BPs) known to play a role in the disease, including apoptosis, cell adhesion, immune response, type I Interferon signaling, Interferon-gamma signaling, extracellular matrix (ECM) organization and morphogenesis of a branching epithelium. A selection of genes that play a role in the above mentioned BPs is shown in Table 1.

LncRNA profiling showed a statistically significant variation (Bonferroni-corrected *p*-value ≤  0.01 and fold change  ≥ |1.5|) for 199 long non-coding RNAs (Table S2).

Since modulated genes are well representative of BPs and pathways strictly connected to the disease, we decided to verify whether the modulated lncRNAs could be connected functionally to the pSjS transcriptome, thus playing a role in pSjS pathophysiology.

The identified 199 lncRNAs were then analysed, retaining only those transcripts for which experimentally validated microRNA (miRNA) targets had already been annotated in starBase and, among these, only lncRNAs that targeted at least 10 miRNAs were selected.

Using this approach, we obtained 6 lncRNAs, including CTD-2020K17.1, LINC00657, RP11-169K16.9, LINC00511, RP11-372K14.2 and RP11-214O1.2. (Table 2)

To strengthen the significance of our analysis we wanted to verify the ability of the selected lncRNAs to regulate genes that are differentially expressed in pSjS samples through the interaction with their miRNA targets. We therefore evaluated the entire lists of their miRNA targets that were validated experimentally by high-throughput technologies and selected only those miRNAs that targeted genes modulated in pSjS patients to bona fide outline authentic interactions that are well established in pSjS.

Shown in Table 2, LINC00657, LINC00511 and CTD-2020K17.1 targeted the highest number of modulated genes (Table S3). The expression data were confirmed by RT-PCR (Figure S1).

### 3.3. PPI Network and Modular Analysis of Genes and lncRNAs Modulated in pSjS

Since the modulation of highly connected genes can have a more pronounced effect in the disease pathogenesis than the modulation of genes that are not functionally connected, we evaluated whether the 6 selected lncRNAs could target highly interacting genes in pSjS.

**Figure 1:**
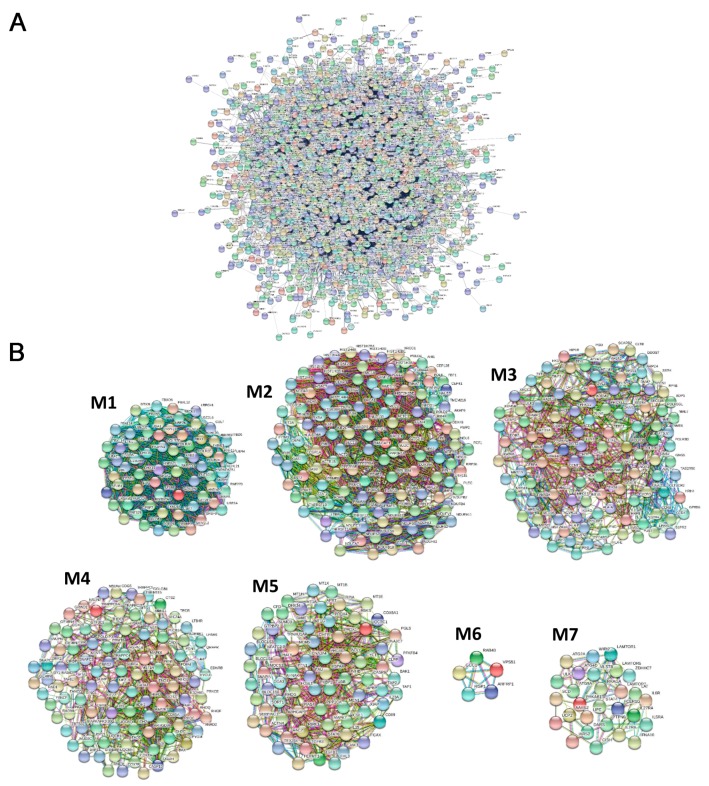
Network analysis of differentially expressed genes (DEGs) in pSjS patients. (**A**) Protein–protein interaction (PPI) network of DEGs; (**B**) Modules originated from the interaction network.

Considering this, to prioritize transcripts that may have a role in the pSjS pathogenesis, we performed a network analysis dissecting all the differentially expressed genes in pSjS that were connected functionally. We therefore constructed a protein–protein interaction (PPI) network that included all the experimentally validated functional interactions among the protein products of the 2503 modulated genes in pSjS (Figure 1A). The obtained network included 2500 modulated genes (nodes) that were connected by 14,169 pairs of interactions (edges) and showed a good PPI enrichment *p*-value (<10^−16^).

Moreover, since genes that are strictly connected to the disease phenotype display a strong tendency to cluster together in few network regions [13], we performed a modular analysis to find areas in which the most highly connected genes were clustered. Using this approach, we could identify 7 gene modules that are most likely to be involved in the disease pathogenesis (Figure 1B and Table S4).

All 7 modules were imported in Cytoscape adding to their scaffolds miRNAs-genes, and lncRNAs—miRNAs interactions.

The topological analysis of such implemented modules highlighted, for the 6 lncRNAs, the lncRNA–gene interactions mediated by their targeted miRNAs. We observed that LINC00657, LINC00511 and CTD-2020K17.1 targeted highly connected genes and the highest number of module-associated genes that were distributed in all the 7 modules (Table 2 and Table S5).

**Figure 2:**
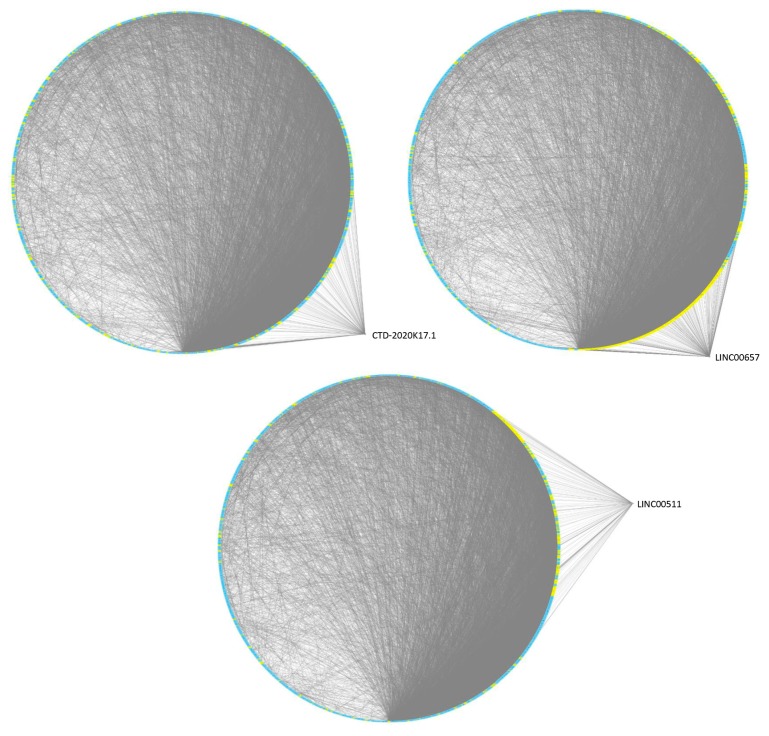
Functional interactions among the three selected lncRNAs and genes modulated in pSjS patients. Degree sorted circle layouts of the protein–protein interaction (PPI) network of differentially expressed genes in pSjS patients are shown. Genes (nodes) are ordered around a circle based on their degree of connectivity (number of edges).

We therefore narrowed our analysis to CTD-2020K17.1, LINC00511, and LINC00657 lncRNAs, since they most probably had a major impact on pSjS transcriptome (as also suggested by the network analysis), (Figure 2). 

Noteworthy, targeted genes included several important transcripts involved in T cell development (GATA3), in the response to type I interferon (IRF5, IRF9 and KLHL20), in inflammatory response (IL6R and CEBPD) and in B-cell physiology and malignancy (BAK1, BAX, CBX8, ENO1, GNAI2, HNRNPL, LTBR, TRAF3 and WDR5).

When we analysed the list of miRNAs regulated by the 3 lncRNAs (Table S6), we found that 6 miRNAs played a role in B cell development, for example mir-17-5p, mir-20b-5p, mir-34a-5p, mir-34c-5p, mir-155-5p and mir-93-5p [14,15], and that 51 miRNAs have been previously associated with several types of human B cell lymphomas. Noteworthy, 15 of the above mentioned miRNAs were already associated to pSjS (see Table 3).

### 3.4. Functional Analysis of Modulated Genes Targeted in the Whole Transcriptome

Once we identified the three lncRNAs that most likely exert a major impact on the whole transcriptome, we wanted to verify if these transcripts could regulate genes that may play a crucial role in the disease. 

The three lncRNAs targeted genes involved in several meaningful biological processes. Indeed, LINC00657 regulated a large number of genes involved in cell adhesion including HN1 (F.C. +2.71), ITGA5 (F.C. +2.22), MISP (F.C. +1.86), PRKCE (F.C. −1.62), PTPRJ (F.C. +1.71), RHOC (F.C. +2.5), ZEB1 (F.C. −4.18) and ZFYVE21 (F.C. +3.07) as well as several transcripts that play a role in epithelial cell polarization such as ARF6 (F.C. +1.53), FRMD4A (F.C. +1.859) and RHOQ (F.C. +1.79). Additionally, many genes implicated in apoptosis also were targeted by LINC00657, including ANP32B (F.C. +2.34), BCL2L12 and BCL2L13 (F.C. +2.44 and +2.49 respectively), BMF (F.C. +1.88), BRI3 (F.C. +1.87), CLPTM1L (F.C. +2.8) and ING2 (F.C. +1.97). We also observed that LINC00657 regulated genes involved in T cell development (GATA3, F.C. +1.74) and in T cell activation (ORAI1, F.C. +2.97) as well as genes related to B cell activity, i.e., CBX8 (F.C. +1.62), ENO1 (F.C. +1.91), GNAI2 (F.C. +1.67), SPI1/PU.1 (F.C. +1.73) and TRAF3, a critical regulator of B cell homeostasis (F.C. −1.63). Finally, LINC00657 targeted genes involved in the inflammatory response including CEBPD (F.C. +1.94), LTB4R (F.C. +2.46), MAP3K12 (F.C. +1.88) and (TRIB2 (F.C. +2.19) and in type I interferon signaling such as IRF5 (F.C. +2.24), IRF9 (F.C. +2.68) and KLHL20 (F.C. −1.94).

LINC00511 regulated a large number of transcripts involved in apoptosis, including BMF (F.C. +1.88), BFAR (F.C. 1.98), DAP (F.C. +2.42) PDCD6 (F.C. +2.93), SORT1 (F.C. +2.51) and RRAGA (F.C. +1.76) whereas CTD-2020K17.1 predominantly targeted differentially expressed genes involved in B cell functions including GNAI2 (F.C. +1.67) HNRNPL (F.C. +2.16) FOSL1/FRA1, the inhibitor of follicular B cell differentiation into plasma cells (F.C. −2.14) and the previously mentioned TRAF3. Moreover, CTD-2020K17.1 also regulated the BAK1 gene (F.C. +2.46) that is frequently overexpressed in B cell lymphomas.

Since it is widely acknowledged that disease phenotypes reflect complex molecular interactions and that human disorders should be considered in terms of perturbations of molecular interaction networks [13], we next performed a pathway enrichment analysis of genes targeted by the three selected lncRNAs.

**Figure 3:**
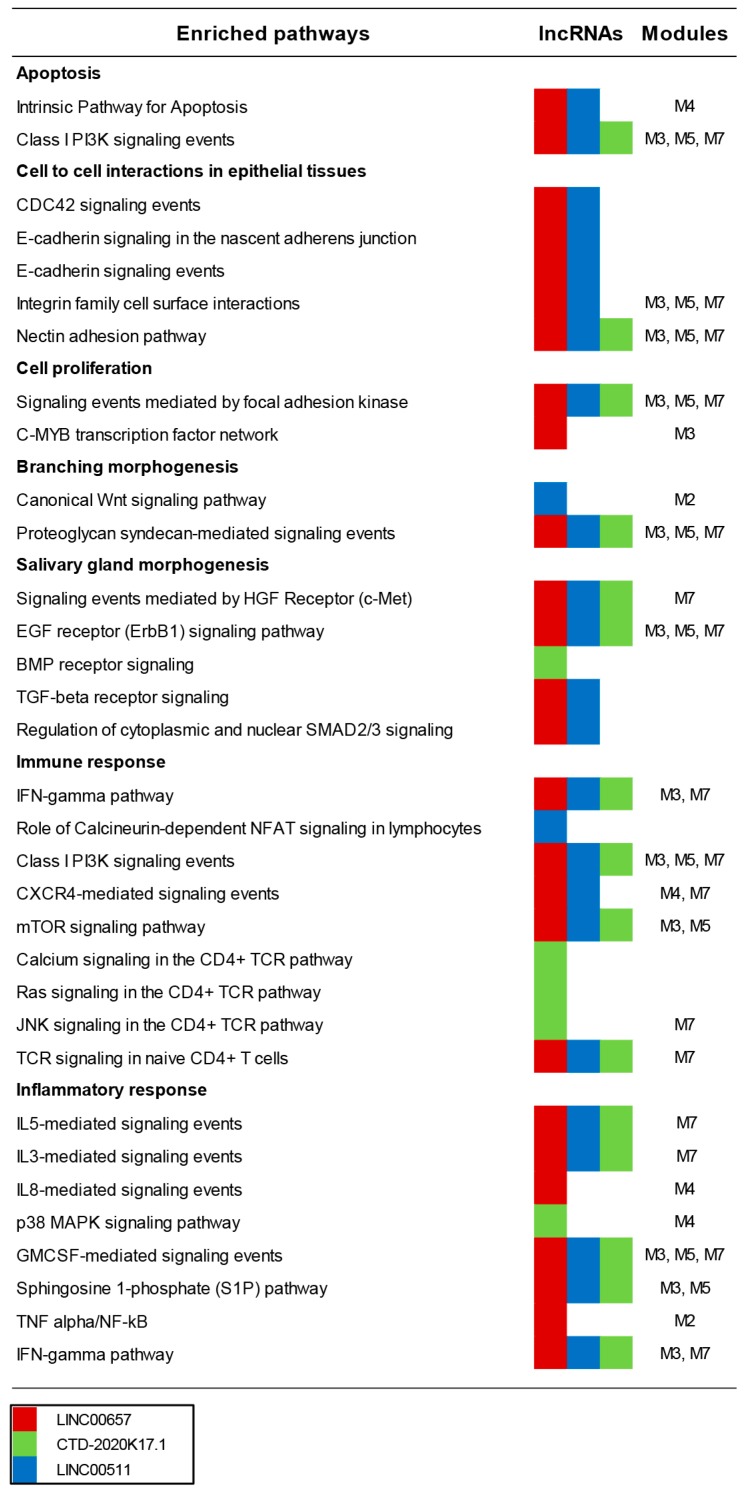
Meaningful pathways enriched in modulated genes targeted by the three selected lncRNAs. Colored squares indicate pathways enriched in genes targeted by the three lncRNAs: CTD-2020K17.1 (green squares); LINC00657 (red squares); LINC00511 (blue squares). The modules with the same enriched pathways are shown.

Signaling pathways involved in apoptosis were enriched in genes targeted by the three lncRNAs which included “Intrinsic Pathway for Apoptosis” (LINC00511, and LINC00657) and “Class I PI3K signaling events mediated by Akt” (CTD-2020K17.1, LINC00511, and LINC00657) (Figure 3 and Table S7).

Several targeted pathways that play a role in cell-to-cell interactions in epithelial tissue were enriched including: “CDC42 signaling events” (LINC00511 and LINC00657); “E-cadherin signaling events” (LINC00511 and LINC00657); “E-cadherin signaling in the nascent adherens junction” (LINC00511 and LINC00657); “Integrin family cell surface interactions” (LINC00511 and LINC00657); and “Nectin adhesion pathway” (CTD-2020K17.1, LINC00511 and LINC00657). 

Signaling pathways involved in cell proliferation also were enriched i.e., “Signaling events mediated by focal adhesion kinase” (CTD-2020K17.1, LINC00511 and LINC00657) and “C-MYB transcription factor network” (LINC00657). Moreover, pathways implicated in branching morphogenesis were targeted including “Canonical Wnt signaling pathway” (LINC00511) and “Proteoglycan syndecan-mediated signaling events” (CTD-2020K17.1, LINC00511 and LINC00657). Interestingly, three pathways regulating salivary gland morphogenesis were targeted by the selected lncRNAs and these encompassed “EGF receptor signaling pathway” (CTD-2020K17.1, LINC00511 and LINC00657), “Signaling events mediated by Hepatocyte Growth Factor Receptor (c-Met)” (CTD-2020K17.1, LINC00511 and LINC00657) and “BMP receptor signaling” (CTD-2020K17.1). “TGF-beta receptor signaling”, a pathway that influences salivary gland physiology, and its downstream pathway “SMAD signaling” were targeted by LINC00511 and LINC00657. 

Several pathways ascribed to the immune response were enriched in modulated targeted genes including “IFN-gamma pathway” (CTD-2020K17.1, LINC00511 and LINC00657), “CD4+ TCR pathway” (CTD-2020K17.1, LINC00511 and LINC00657) and “Role of Calcineurin-dependent NFAT signaling in lymphocytes” (LINC00511). Particularly, several pathways involved in B cell development and activation were enriched including the previously mentioned “Class I PI3K signaling events mediated by Akt” (CTD-2020K17.1, LINC00511, and LINC00657), “CXCR4-mediated signaling events” (LINC00511 and LINC00657), and the mTor signaling (CTD-2020K17.1, LINC00511 and LINC00657). 

The three lncRNAs targeted several proinflammatory pathways such as “Interleukin-3” (CTD-2020K17.1, LINC00511 and LINC00657), “Intreleukin-5” (CTD-2020K17.1, LINC00511 and LINC00657) and “Interleukin-8” (LINC00657) signaling, “p38 MAPK signaling pathway” (CTD-2020K17.1), “GMCSF-mediated signaling events” (CTD-2020K17.1, LINC00511 and LINC00657), “Sphingosine 1-phosphate (S1P) pathway” (CTD-2020K17.1, LINC00511 and LINC00657) “TNF alpha/NF-kB” (LINC00657) and the above cited “IFN-gamma pathway” (Figure 3).

### 3.5. Functional Analysis of Highly Connected Genes that are Included in Modules

Since genes that show the highest number of interactions in the transcriptome most likely are involved in the disease pathogenesis, we next performed a functional analysis of the seven identified gene modules.

**Figure 4:**
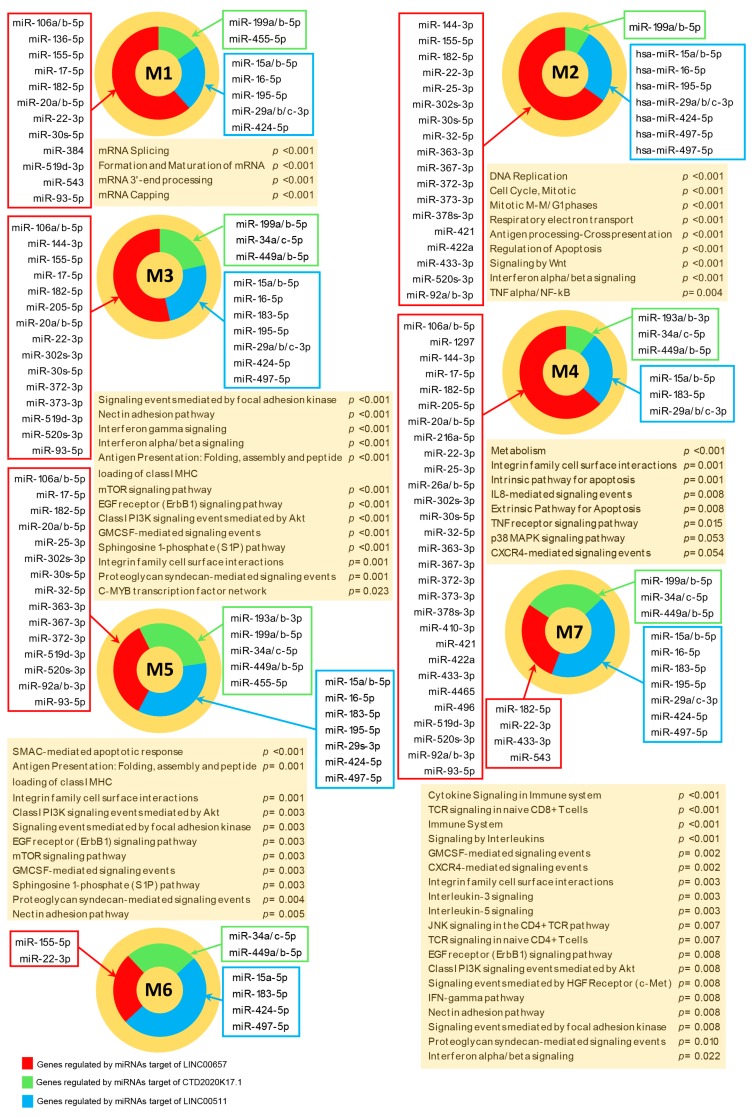
Pathways enrichment of modulated genes included in the modules. Orange circles represent the seven modules and selected enriched pathways in each module are listed in the orange boxes along with their relative statistical significance (*p*-value). Colored pie-charts indicate the percentage of genes targeted in each module by CTD-2020K17.1 (green); LINC00657 (red) and LINC00511 (blue). Colored boxes indicate miRNAs that target genes included in the modules and that are targeted by CTD-2020K17.1 (green box), LINC00657 (red box) and LINC00511 (blue box).

Figure 4 summarizes genes targeted in the 7 modules and their respective targeting miRNAs along the most relevant enriched signaling pathways.

M1 included 66 genes and was globally targeted by 29 miRNAs. The top enriched (*p* ≤ 0.05) pathways in Module M1 were those involved in mRNA processing, including mRNA splicing (Table S8).

M2, included 123 genes and was targeted by 49 miRNAs. Aside from pathways related to cell division (i.e., “DNA Replication”, “Cell Cycle, Mitotic” and “Mitotic M-M/G1 phases”) and energy metabolism (i.e., “Respiratory electron transport”) other meaningful pathways were enriched in this module, including signaling of Interferon alpha/beta and gamma, “Antigen processing-Cross presentation”, “Regulation of Apoptosis”, “Signaling by Wnt” and “TNF alpha/NF-kB” signaling.

M3 included 108 genes and was targeted by 45 miRNAs. “Interferon gamma”, “Interferon alpha/beta”, “Antigen Presentation: Folding, assembly and peptide loading of class I MHC”, “mTOR signaling pathway”, “Class I PI3K signaling events mediated by Akt”, “EGF receptor (ErbB1) signaling pathway” and “GMCSF-mediated signaling events” were among the most relevant enriched pathways in Module M3.

M4 included 101 genes and was the most targeted module, indeed 64 miRNAs were found to be linked to it. This module mostly was enriched in pathways involved in cell metabolism and in other important pathways including “Extrinsic Pathway for Apoptosis”, “IL8-mediated signaling events”, “TNF receptor signaling pathway”, “p38 MAPK signaling pathway” and “CXCR4-mediated signaling events”.

M5 included 74 genes and was targeted by 49 miRNAs. Pathways related to apoptosis were the most enriched in module M5, aside from the “Antigen Presentation” pathway. Other important pathways highly enriched in this module also included “Class I PI3K signaling events mediated by Akt”, “EGF receptor (ErbB1) signaling pathway”, mTOR signaling pathway” and “GMCSF-mediated signaling events”.

M6 included five genes and was targeted by 10 miRNAs. We did not observe any statistical enrichment in Module M6, which was probably due to the small number of genes that composed this cluster.

Finally, M7 included 28 genes and was targeted by 20 miRNAs. “Cytokine Signaling in Immune system”, “TCR signaling in naive CD8+ T cells” and “Immune System” were the most enriched signaling pathways in Module M7. Additional notable pathways enriched in this module were “Signaling by Interleukins”, “GMCSF-mediated signaling events”, “CXCR4-mediated signaling events”, “EGF receptor (ErbB1) signaling pathway”, “Class I PI3K signaling events mediated by Akt”, “Signaling events mediated by Hepatocyte Growth Factor Receptor (c-Met)”, “IFN-gamma pathway” and “Interferon alpha/beta signaling”.

When we performed a pathway enrichment analysis on the genes targeted within the 7 modules, we found that most of the pathways enriched in the modules also were enriched in the genes targeted in the whole transcriptome. This finding suggests that regulation of highly interactive genes associated with the modules may have a prominent role in the pSjS transcriptome.

## 4. Discussion

Sjögren’s syndrome is a chronic autoimmune disorder, primarily affecting exocrine glands, and is of unknown origin. The interplay among genetic background, environmental factors, mainly infectious agents, and epigenetics, however, play a pivotal role on the pathogenesis of the disease.

Long non-coding RNAs (lncRNAs) exert a control on gene expression at multiple levels and recently have emerged as crucial components of the epigenetic machinery. Moreover, a growing body of evidence has highlighted the involvement of lncRNAs in different types of autoimmune diseases [47,48].

Several disturbed epigenetic mechanisms, including DNA demethylation, microRNAs modulation and aberrant chromatin positioning have been described in pSjS [49]; however, very little is known of the potential role played by lncRNAs in pSjS. Particularly, a detailed analysis of functional interactions among lncRNA and the pSjS transcriptome has not been attempted yet. During this study we have, therefore, inspected crucial molecular interactions among modulated genes and lncRNAs in pSjS, interactions that may be of relevance in the pathogenesis of the disease.

The expression profiles of a vast number of coding and non-coding genes have been analysed at the same time and, by a multiple step process, modulated lncRNAs that possibly were connected to the pSjS transcriptome were selected.

A sophisticated network analysis was carried out on the entire set of molecular interactions among modulated genes and lncRNAs, thus outlining reliable lncRNA–miRNA–gene functional interactions.

Through a complex network analysis of the whole set of molecular interactions among modulated genes and lncRNAs, we further selected those lncRNAs that targeted modules of the most highly connected genes in the pSjS interactome, since they may have a major impact on pSjS gene modulation. Using this procedure, we chose lncRNAs on the basis of their connectivity (highly versus poorly connected lncRNAs).

Using this approach, we identified three lncRNAs, namely LINC00657, LINC00511 and CTD-2020K17.1, characterized by a high degree of connectivity. Considering this, LINC00657 could exert a prominent role in pSjS gene modulation since it targeted the highest number of highly connected transcripts and, therefore, regulated the vast majority of the disease-related pathways.

Noteworthy, many of the miRNAs targeted by these three lncRNA, which modulate highly connected genes, have been already associated to pSjS.

Several targeted genes with a high degree of connectivity were involved in inflammation, such as the IL-6 receptor (IL6R) and the enhancer of IL-6 production (CEBPD) or in the immune response, such GATA3, that is crucial for T cell development, and the three type I interferon-responsive-genes IRF5, IRF9 and KLHL20.

Genes modulated during type I interferon pathway activation (like IRF5 and STAT4) were associated with risk of pSjS, and a type I interferon signature has been described in pSjS patients. Moreover, this signature strongly correlates with the presence of anti-SSA/Ro antibodies [50].

Interestingly, the three lncRNAs also targeted highly connected genes involved in B-cell physiology and malignancy, including IL15 (LINC00657), WDR5 (LINC00657), GNAI2 (LINC00657 and CTD-2020K17.1), LTBR (LINC00511), CBX8 (LINC00657), BAK1 (CTD-202K17.1 and LINC00511), BAX (LINC00511), ENO1 (LINC00657), HNRNPL (CTD-2020K17.1), and TRAF3 (LINC00657 and CTD-2020K17.1).

The involvement of B cells in the pSjS pathogenesis has been documented well and an increased number of mature B cells have been found in patients with pSjS [51]; interestingly, IL15 and WDR5 play a crucial role in both B cell proliferation and differentiation and GNAI2 regulates B cell trafficking into and within lymph nodes [52]. Moreover, germinal centre (GC)-like structures have been described within the pSjS salivary gland epithelium [51] and, not surprising, LINC00511 and LINC00657 respectively targeted the modulated genes LTBR and CBX8 that are involved on GC formation in inflamed tissues [53,54]. Worth mentioning, three patients presented germinal centre-like structures in their labial salivary gland biopsy.

It has been observed that pSjS patients have an increased risk to develop B cell lymphoproliferative disorders [55] and, noteworthy, BAK1 and BAX are overexpressed in diffuse large B cell lymphoma [56]; ENO1 promotes tumor proliferation in Non-Hodgkin’s Lymphomas and stimulates immunoglobulin production [57]; hnRNPL induces BCL2 overexpression in many B cell lymphomas [58] whereas TRAF3 is a tumor suppressor gene in B lymphocytes and frequently is inactivated in human B lymphoma and multiple myeloma [59]. Interestingly, we found that TRAF3 resulted in down-modulation in our patients with pSjS.

We also observed that several miRNAs targeted by the three lncRNAs that modulated highly connected genes have a documented role in B cell development and in B cell lymphomagenesis. It is worthwhile mentioning that one of the patients developed a B cell lymphoma soon after enrollment in the study.

It has been long recognised that disorders can be viewed in the context of signaling pathway deregulation. We therefore inspected all the signaling pathways that were enriched in modulated genes targeted by the three selected lncRNAs and we discovered that they regulate well-known signaling networks. Moreover, we observed that the vast majority of these pathways also were enriched in all the seven targeted gene modules, thus indicating that the three lncRNAs targeted pathways closely related to the disease pathogenesis.

The selected lncRNAs targeted pathways involved in the development of lacrimal and salivary glands including those of Syndecan (LINC00657, LINC00511 and CTD-2020K17.1), Wnt (LINC00511), TGF-beta receptor (LINC00657 and LINC00511), BMP receptor (CTD-2020K17.1), SMAD proteins (LINC00657 and LINC00511), EGF receptor (LINC00657, LINC00511 and CTD-2020K17.1) and HGF receptor (LINC00657, LINC00511 and CTD-2020K17.1).

The development of both salivary and lacrimal glands is a finely tuned process defined as “branching morphogenesis” that relies on the differential expression of cellular surface receptors and extracellular matrix components like proteoglycans. Indeed, the interactions between cell receptors and ECM elements boost the activation of downstream intracellular signaling pathways that induce the differentiation of progenitor cells into acinar lobules and ducts [60].

Among these pathways, the Wnt signaling is involved in distinct space and temporal patterns during salivary gland development [61] and, interestingly, hypermethylation of this pathway has been observed in salivary gland epithelial cells from patients with Sjögren’s syndrome [62].

TGF-beta also can control salivary gland development by modulating cellular growth and differentiation, moreover, it alters salivary gland physiology by regulating angiogenesis and inflammation [63]. Noteworthy, it has been observed that deletion of TGF-beta signaling in mice salivary glands induces a Sjögren’s syndrome-like autoimmune disorder [63].

The Smad and BMP proteins are downstream mediators of the TGF-beta signaling and influence branching morphogenesis of both lacrimal and salivary glands by promoting mesenchymal proliferation [64,65]. Interestingly, through Smad proteins, TGF-beta also stimulates transcription and subsequent IgA isotype expression in B lymphocytes [66].

Another signaling pathway involved in salivary gland morphogenesis is that of EGF [67], a molecule that also is secreted by lacrimal glands and represents one of the most abundant growth factors in human tears [68]. Particularly, this cytokine can boost migration and proliferation of corneal epithelial cells during wound healing [68].

HGF plays a crucial role in salivary gland development [69] and modulates cell behaviour in acinar and ductal epithelial cells of lacrimal glands [70].

It is well known that the structural and morphological integrity of cell aggregates is pivotal to maintain epithelial functional homeostasis and one of the crucial events involved in the development of autoimmunity is the loss of the mucosal barrier integrity, as in the case of Sjögren’s syndrome, where the protective function of glandular epithelia is compromised [71].

Interestingly, we observed that the three selected lncRNAs target molecular pathways that regulate cell adhesion, for example those of integrins (LINC00657 and LINC00511), E-cadherins (LINC00657 and LINC00511), Nectins (LINC00657, LINC00511 and CTD-2020K17.1), CDC42 (LINC00657 and LINC00511) and focal adhesion kinases (LINC00657, LINC00511 and CTD-2020K17.1).

Marked alteration of integrin expression has been described in pSjS salivary glands [72] and it has been related to the observed detachment of glandular acinar cells. Moreover, elevated serum levels of the soluble form of the adherens junction-associated proteins, E-cadherins, were detected in pSjS patients and it has been related to tissue regeneration induced by chronic inflammation in pSjS glandular epithelia [71]. Additionally, E-cadherins also play a pivotal role in the adhesive interactions between epithelial cells and intraepithelial lymphocytes [73].

To form adherens junctions, E-cadherins associate to the nectin proteins that also are involved in the formation of tight junctions [74]. Specifically, nectins induce activation of Cdc42-mediated signaling, regulating cell polarization and, noteworthy, it has been observed that the secretory dysfunction in pSjS patients also is due to the impairment of epithelial cell polarity [75].

The Cdc42 molecules, in turn, also modulate the activation state of the focal adhesion kinase proteins, another junctional system deregulated in pSjS [71].

Severe disaggregation of the basal lamina associated to the glandular epithelium has been described in pSjS and this event allows inflammatory cells and cytotoxic T lymphocytes to infiltrate acini and ducts [76]. Proteoglycans are a key component of the basal lamina structure and, interestingly, all three lncRNAs targeted proteoglycans, signaling suggesting that they may also be involved in the basal lamina modification, a key feature in pSjS pathogenesis.

Aside from the above mentioned histopathological changes, apoptosis also is involved in pSjS-associated glandular damage, and both apoptotic cells and expression of the apoptosis-regulating proteins have been detected in salivary glands from pSjS patients in many studies [77]. Particularly, it has been suggested that apoptosis might be the initial trigger for autoimmunity in this disease [76] and, noteworthy, proapoptotic signaling pathways also were targeted by the selected lncRNAs.

Leukocyte infiltration in the damaged pSjS glandular epithelium enhances the inflammatory environment that also is sustained by epithelial cell production of adhesion molecules and chemokines [78]. Several proinflammatory pathways were targeted by the selected lncRNAs, including signaling by TNF/NF-Kb (LINC00657), p38 MAP kinase (CTD-2020K17.1), IFN-gamma (LINC00657, LINC00511 and CTD-2020K17.1), GMCSF (LINC00657, LINC00511 and CTD-2020K17.1) and IL-8 (LINC00657).

Tumor Necrosis Factor (TNF) is a strong inducer of NF-kappa B transcription [79] that has been implicated in the development of several autoimmune diseases, and correlates to the chronic inflammation that characterizes pSjS [80]. Moreover, the proinflammatory factor p38 MAP kinase is thought to be involved in the pathogenesis of pSjS [81]. Sphingosine-1-phosphate regulates T cell development and tissue-homing patterns and plays an important role in the local immune responses in pSjS glandular tissue [82]. Moreover, together with IFN-gamma, this molecule is thought to increase IL-6 secretion by pSjS salivary glands [82].

IFN-γ is crucial for T cell differentiation and immunoglobulin class switching in B cells and plays an important role in macrophage activation, inflammation and tissue damage [83]. Moreover, this molecule has been implicated in the development of autoimmunity as well as being associated with more severe forms of pSjS [84].

The immune system has a crucial role in the tissue damage observed in pSjS and both innate and adaptive immunity strongly contribute to this event. Specifically, it has been described that the type-I interferon (IFN-I) signature is involved crucially in the disease pathogenesis [85] and, interestingly, the selected lncRNAs targeted gene modules involved in IFN-I signaling, moreover, they also targeted molecular pathways of the T cell-associated immune response (i.e., TCR and calcineurin-dependent NFAT signaling).

It has been observed that T cells represent the majority of lymphocytes infiltrating the pSjS salivary glands and pro-inflammatory Th1 cell cytokines are increased in saliva of patients with pSjS [86]. Additionally, the presence of regulatory T cells (Treg) also have been identified in pSjS salivary glands and their increased presence is related strongly to a high rate of inflammation in tissue lesions [86]. Noteworthy, the calcineurin-dependent NFAT signaling plays a pivotal role for both development and functions of these cells [87].

There is a body of evidence also supporting the involvement of B cells in pSjS pathogenesis and, indeed, pSjS patients clearly manifest clinical signs of B cell activation [51]. Furthermore, B cells have been identified in the pSjS salivary glands where they can form ectopic germinal centres [51]. As we previously mentioned, we describe that selected lncRNAs can modulate miRNAs involved in B cell development and, interestingly, we also observed that they targeted signaling pathways involved in B cell differentiation and functions such as CXCR4 (LINC00657 and LINC00511) signaling, Class I PI3K and mTor pathways (LINC00657, LINC00511 and CTD-2020K17.1).

Class I PI3K is expressed predominantly by B cells and its signaling pathway is activated promptly upon B cell receptor engagement [88]. Moreover, inhibition of this pathway strongly interferes with pre-B-cell expansion [89]. The PI3k signaling also activates the Akt and mTor pathways which also are involved in B cell proliferation [88] and, noteworthy, their activation has been observed in B cell lymphomas [88]. Finally, an important role for CXCR4 in regulating B cell homeostasis of humoral immunity has been described [90] and noteworthy, a significantly higher expression of both surface CXCR4 and CXCR4 messenger RNA (mRNA) has been reported in peripheral blood B cells from patients with pSjS [91].

We are aware of the limitations of gene expression and microarray analyses, limitations both at preanalytical and analytical levels. Thus, the first, and most important, aspect at a preanalytical level is the correct stratification of the patients and the identification of subjects at the same stage of disease and with similar clinical features. Another important aspect is that the analysis reflects a particular moment in the course of the disease. The method we are reporting here, however, is the most informative for a global evaluation of gene expression, microRNA and lncRNA and, therefore, for the understanding of the genetic and epigenetic processes that play a pivotal role in the pathogenesis of immune-mediated diseases.

## 5. Conclusions

To conclude, this is the first report that functionally correlates lnc-RNAs modulation to gene expression profiles of pSjS patients. Indeed, the described lncRNAs target gene pathways that are involved in important features of the disease, including epithelial cell damage, autoimmunity and B cell hyperactivation. Furthermore, the selected lncRNAs regulate B cell lymphomagenesis-associated microRNAs, thus suggesting their possible involvement in the increased risk to develop lymphoma that is observed in pSjS patients.

Taken together, our findings add new pieces of evidence on the importance of epigenetics in the pathogenesis of pSjS and may open promising avenues for novel therapeutic approaches to the treatment of the disease.

## Figures and Tables

**Table 1:** Selection of significant differentially expressed genes in pSjS patients versus healthy subjects, grouped according to the biological processes to which they are ascribed.

Gene Symbol	Description	Fold Change	*p*-Value	mRNA Accession
**Apoptosis**				
CCAR2	cell cycle and apoptosis regulator 2	1.83	0.002	NM_021174
CASP10	caspase 10	2.26	0.004	NM_001206524
BCL2L13	BCL2-like 13 (apoptosis facilitator)	2.49	<0.001	NM_001270726
BCL2L12	BCL2-like 12 (proline rich)	2.44	0.002	NM_001040668
PYCARD	PYD and CARD domain containing	2.52	0.004	NM_013258
DAPK3	death-associated protein kinase 3	2.09	<0.001	NM_001348
PDCD6	programmed cell death 6	2.93	<0.001	NM_001267556
CASP9	caspase 9	2.01	<0.001	NM_001229
DEDD2	death effector domain containing 2	3.82	<0.001	NM_001270614
BMF	Bcl2 modifying factor	1.88	0.003	NM_001003940
PDCD7	programmed cell death 7	1.98	0.001	NM_005707
FADD	Fas (TNFRSF6)-associated via death domain	2.06	<0.001	NM_003824
ANP32B	acidic nuclear phosphoprotein 32 family member B	2.34	<0.001	NM_006401
BRI3	brain protein I3	1.87	0.003	NM_001159491
CLPTM1L	CLPTM1-like	2.8	<0.001	NM_030782
ING2	inhibitor of growth family member 2	1.97	<0.001	NM_001291959
BFAR	bifunctional apoptosis regulator	1.98	0.003	NM_016561
DAP	death-associated protein	2.42	0.004	NM_001291963
SORT1	sortilin 1	2.51	0.008	NM_001205228
RRAGA	Ras-related GTP binding A	1.76	0.001	NM_006570
**Cell adhesion**				
LIN7C	lin-7 homolog C (C. elegans)	−1.76	0.001	NM_018362
ITGB3BP	integrin beta 3 binding protein (beta3-endonexin)	−2.87	0.001	NM_001206739
PRKCE	protein kinase C, epsilon	−1.62	0.005	NM_005400
FER	fer (fps/fes related) tyrosine kinase	−2.3	0.001	NM_001308028
ADAM8	ADAM metallopeptidase domain 8	3.13	<0.001	NM_001109
ADAM15	ADAM metallopeptidase domain 15	3.59	<0.001	NM_001261464
CLDN5	claudin 5	1.69	0.007	NM_001130861
EMP2	epithelial membrane protein 2	1.71	0.005	NM_001424
ICAM3	intercellular adhesion molecule 3	2.86	<0.001	NM_002162
HN1	hematological and neurological expressed 1	2.71	<0.001	NM_001002032
ITGA5	integrin alpha 5	2.22	<0.001	NM_002205
MISP	mitotic spindle positioning	1.86	0.003	NM_173481
PTPRJ	protein tyrosine phosphatase, receptor type, J	1.71	0.006	NM_001098503
RHOC	ras homolog family member C	2.5	0.004	NM_001042678
ZEB1	zinc finger E-box binding homeobox 1	−4.18	<0.001	NM_001128128
ZFYVE21	zinc finger, FYVE domain containing 21	3.07	0.001	NM_001198953
**Immune response**				
LAMTOR2	late endosomal/lysosomal adaptor, MAPK and MTOR activator 2	3.08	0.002	NM_001145264
IL6R	interleukin 6 receptor	1.79	0.001	NM_000565
IGKV3D-7	immunoglobulin kappa variable 3D-7	4.17	0.004	OTTHUMT00000476805
IGLV3-19	immunoglobulin lambda variable 3-19	6.58	0.002	OTTHUMT00000321830
HLA-G	major histocompatibility complex, class I, G	2.98	<0.001	NM_002127
NCF1	neutrophil cytosolic factor 1	2.17	0.003	NM_000265
CD68	CD68 molecule	2.18	0.002	NM_001040059
DEF6	DEF6 guanine nucleotide exchange factor	1.58	0.003	NM_022047
LILRA6	leukocyte immunoglobulin-like receptor, subfamily A, member 6	2.37	0.004	NM_024318
TRBV24-1	T cell receptor beta variable 24-1	3.57	0.001	OTTHUMT00000352499
IL5RA	interleukin 5 receptor, alpha	2.14	<0.001	NM_000564
CD6	CD6 molecule	3.71	<0.001	NM_001254750
CD7	CD7 molecule	1.93	<0.001	NM_006137
BTK	Bruton agammaglobulinemia tyrosine kinase	1.79	0.006	NM_000061
BAX	BCL2-associated X protein	3.14	<0.001	NM_001291428
BAK1	BCL2-antagonist/killer 1	2.46	0.005	NM_001188
GATA3	GATA binding protein 3	1.74	0.008	NM_001002295
TAP1	antigen peptide transporter 1	2.58	<0.001	NM_001292022
TAP2	antigen peptide transporter 2	2.23	0.002	NM_018833
IL17RA	interleukin 17 receptor A	2.52	0.001	NM_001289905
IL23A	interleukin 23, alpha subunit p19	−2.9	0.009	NM_016584
IL4R	interleukin 4 receptor	2.37	<0.001	NM_000418
CD33	CD33 molecule	3.39	0.002	NM_001082618
IL2RA	interleukin 2 receptor, alpha	1.89	0.006	NM_000417
LAT	linker for activation of T-cells	3.25	<0.001	NM_001014987
C1RL	complement component 1, r subcomponent-like	1.52	0.010	NM_001297640
FCGR1A	Fc fragment of IgG, high affinity Ia, receptor (CD64)	3.2	<0.001	NM_000566
CD81	CD81 molecule	2.04	0.008	NM_001297649
KLHL20	kelch-like family member 20	−1.94	0.004	NM_014458
KLHL6	kelch-like family member 6	2.18	0.005	NM_130446
ORAI1	ORAI calcium release-activated calcium modulator 1	2.97	<0.001	NM_032790
CBX8	chromobox homolog 8	1.62	0.008	NM_020649
ENO1	enolase 1, (alpha)	1.91	0.004	NM_001201483
GNAI2	guanine nucleotide binding protein, alpha inhibiting activity polypeptide 2	1.67	0.007	NM_001166425
SPI1	Spi-1 proto-oncogene	1.73	0.007	NM_001080547
TRAF3	TNF receptor-associated factor 3	−1.63	0.009	NM_001199427
HNRNPL	heterogeneous nuclear ribonucleoprotein L	2.16	0.001	NM_001005335
FOSL1	FOS-like antigen 1	−2.14	0.008	NM_001300844
**Type I interferon signaling**				
IRF5	interferon regulatory factor 5	2.24	0.003	NM_001098627
IRF7	interferon regulatory factor 7	3.14	0.002	NM_001572
IRF9	interferon regulatory factor 9	2.68	<0.001	NM_006084
MYD88	myeloid differentiation primary response 88	1.86	0.005	NM_001172566
HLA-H	major histocompatibility complex, class I, H (pseudogene)	2.18	0.004	NR_001434
OAS1	2-5-oligoadenylate synthetase 1	3.58	0.002	NM_001032409
IFI35	interferon-induced protein 35	1.78	0.007	NM_005533
IFITM3	interferon induced transmembrane protein 3	2.54	0.006	NM_021034
IFNA10	interferon, alpha 10	1.99	0.002	NM_002171
ADAR	adenosine deaminase, RNA-specific	3.15	0.005	NM_001025107
OAS3	2-5-oligoadenylate synthetase 3	4.27	0.008	NM_006187
STAT1	signal transducer and activator of transcription 1	1.75	0.008	NM_007315
KLHL20	kelch-like family member 20	−1.94	0.004	NM_014458
**Interferon-gamma signaling**				
HLA-G	major histocompatibility complex, class I, G	2.98	<0.001	NM_002127
FCGR1B	Fc fragment of IgG, high affinity Ib, receptor (CD64)	2.25	0.002	NM_001004340
PML	promyelocytic leukemia	3.18	0.002	NM_002675
STAT1	signal transducer and activator of transcription 1	1.75	0.008	NM_007315
TRIM21	tripartite motif containing 21	2.13	0.002	NM_003141
FCGR1A	Fc fragment of IgG, high affinity Ia, receptor (CD64)	3.2	<0.001	NM_000566
**Inflammatory response**				
CCR4	chemokine (C-C motif) receptor 4	3.73	0.005	NM_005508
CCR8	chemokine (C-C motif) receptor 8	2.24	0.004	NM_005201
IL6R	interleukin 6 receptor	1.79	0.001	NM_000565
MYD88	myeloid differentiation primary response 88	1.86	0.005	NM_001172566
CSF1	colony stimulating factor 1 (macrophage)	2.23	0.005	NM_000757
MIF	macrophage migration inhibitory factor (glycosylation-inhibiting factor)	2.48	<0.001	NM_002415
TNFRSF1A	tumor necrosis factor receptor superfamily, member 1A	4.39	<0.001	NM_001065
TGFB1	transforming growth factor beta 1	1.71	0.003	NM_000660
LTB4R	leukotriene B4 receptor	2.46	<0.001	NM_001143919
ALOX5	arachidonate 5-lipoxygenase	2.57	0.007	NM_000698
IL23A	interleukin 23, alpha subunit p19	−2.9	0.009	NM_016584
CXCR3	chemokine (C-X-C motif) receptor 3	2.25	0.002	NM_001142797
MIF	macrophage migration inhibitory factor (glycosylation-inhibiting factor)	2.48	<0.001	NM_002415
CEBPD	CCAAT/enhancer binding protein (C/EBP), delta	1.94	0.007	NM_005195
MAP3K12	mitogen-activated protein kinase kinase kinase 12	1.88	0.005	NM_001193511
TRIB2	tribbles pseudokinase 2	2.19	0.005	NM_021643
**ECM organization**				
MMP9	matrix metallopeptidase 9	3.31	0.002	NM_004994
EFEMP2	EGF containing fibulin-like extracellular matrix protein 2	2.43	0.001	NM_016938
ADAM15	ADAM metallopeptidase domain 15	3.59	<0.001	NM_001261464
ADAM8	ADAM metallopeptidase domain 8	3.13	<0.001	NM_001109
BSG	basigin (Ok blood group)	2.07	0.001	NM_001728
DAG1	dystroglycan 1 (dystrophin-associated glycoprotein 1)	1.57	0.003	NM_001165928
CTGF	connective tissue growth factor	1.54	0.006	NM_001901
TGFB1	transforming growth factor beta 1	1.71	0.003	NM_000660
LOXL3	lysyl oxidase-like 3	2.02	0.002	NM_001289164
SPOCK2	sparc/osteonectin, cwcv and kazal-like domains proteoglycan (testican) 2	2.2	0.006	NM_001134434
**Morphogenesis of a branching epithelium**				
CTSZ	cathepsin Z	2.99	0.002	NM_001336
ILK	integrin linked kinase	1.82	0.001	NM_001014794
DAG1	dystroglycan 1 (dystrophin-associated glycoprotein 1)	1.57	0.003	NM_001165928
EDN1	endothelin 1	−2.17	0.002	NM_001168319
TGFB1	transforming growth factor beta 1	1.71	0.003	NM_000660
GREM1	gremlin 1	1.53	0.009	ENST00000633992
ENG	endoglin	2.42	<0.001	NM_000118
**Epithelial cells polarization**				
ARF6	ADP-ribosylation factor 6	1.53	0.008	NM_001663
FRMD4A	FERM domain containing 4A	1.85	0.003	NM_018027
RHOQ	ras homolog family member Q	1.79	0.005	NM_012249

**Table 2:** Selected long non-coding RNAs modulated in pSjS patients versus healthy subjects.

**Gene Symbol**	**Description**	**Fold Change**	***p*-Value**	**Public Gene IDs**
LINC00657	long intergenic non-protein coding RNA 657	1.8	0.001	NR_027451
LINC00511	long intergenic non-protein coding RNA 511	−2.0	0.008	NR_036488
CTD-2020K17.1	novel transcript, antisense to FMNL1	2.7	0.000	ENST00000585471.1
RP11-169K16.9	uncharacterized LOC729614	1.7	0.008	NR_024279
RP11-214O1.2	uncharacterized protein MGC12916	−2.1	0.010	NR_026880
RP11-372K14.2	novel transcript, antisense to SH3D19	−2.1	0.004	ENST00000603472.1
**Gene Symbol**	**miRNA Targets**	**Total Number of Targeted Modulated Genes**	**Targeted Modules**	**Total Number of Targeted Module-Associated Genes**
LINC00657	67	313	7	75
LINC00511	11	194	7	41
CTD-2020K17.1	11	120	7	25
RP11-169K16.9	13	90	5	15
RP11-214O1.2	12	86	4	13
RP11-372K14.2	12	75	4	12

**Table 3:** miRNA targets of the selected lncRNAs that have been associated to lymphoma and/or pSjS.

**MiRNAs Previously Associated to Lymphoma**		
**lncRNA**	**miRNA Target**	**References**
LINC00657	hsa-miR-106a-5p	Diffuse large B cell lymphoma [16]
LINC00657	hsa-miR-106b-5p	Diffuse large B cell lymphoma [16]
LINC00657	hsa-miR-144-3p	Diffuse large B cell lymphoma [17]
LINC00657	hsa-miR-155-5p	Diffuse large B cell lymphoma [18]
LINC00511	hsa-miR-15a-5p	Diffuse large B cell lymphoma [19]
LINC00511	hsa-miR-15b-5p	Mantle cell lymphoma [20]
LINC00511	hsa-miR-16-5p	Diffuse large B cell lymphoma [21]
LINC00657	hsa-miR-17-5p	Burkitt’s lymphoma [22]
LINC00657	hsa-miR-182-5p	Mantle cell lymphoma [22]
LINC00511	hsa-miR-183-5p	Hodgkin’s lymphoma [23]
LINC00511	hsa-miR-195-5p	Diffuse large B cell lymphoma [24]
CTD-2020K17.1	hsa-miR-199a-5p	Mantle cell lymphoma [25]
CTD-2020K17.1	hsa-miR-199b-5p	Diffuse large B cell lymphoma [26]
LINC00657	hsa-miR-20a-5p	Diffuse large B cell lymphoma [16]
LINC00657	hsa-miR-20b-5p	Mantle cell lymphoma [27]
LINC00657	hsa-miR-22-3p	Diffuse large B cell lymphoma [28]
LINC00657	hsa-miR-26a-5p	Burkitt’s lymphoma [29]
LINC00657	hsa-miR-26b-5p	Burkitt’s lymphoma [29]
LINC00511	hsa-miR-29a-3p	Mantle cell lymphoma [30]
LINC00511	hsa-miR-29b-3p	Mantle cell lymphoma [30]
LINC00511	hsa-miR-29c-3p	Mantle cell lymphoma [30]
LINC00657	hsa-miR-302a-3p	Hodgkin Lymphoma [31]
LINC00657	hsa-miR-302b-3p	Hodgkin’s Lymphoma [31]; diffuse large B cell lymphoma [32]
LINC00657	hsa-miR-302c-3p	Hodgkin’s Lymphoma [31]; mantle cell lymphoma [27]
LINC00657	hsa-miR-30a-5p	Non-Hodgkin’s B cell lymphoma [33]
LINC00657	hsa-miR-30b-5p	Non-Hodgkin’s B cell lymphoma [33]
LINC00657	hsa-miR-30c-5p	Non-Hodgkin’s B cell lymphoma [33]
LINC00657	hsa-miR-30d-5p	Non-Hodgkin’s B cell lymphoma [33]
LINC00657	hsa-miR-30e-5p	Non-Hodgkin’s B cell lymphoma [33]
CTD-2020K17.1	hsa-miR-34a-5p	Diffuse large B cell lymphoma [34]
LINC00657	hsa-miR-363-3p	Mantle cell lymphoma [22]
LINC00657	hsa-miR-372-3p	Mantle cell lymphoma [27]
LINC00657	hsa-miR-373-3p	Mantle cell lymphoma [27]
LINC00657	hsa-miR-378a-3p	Hodgkin’s lymphoma [35]
LINC00657	hsa-miR-378b	Epstein–Barr virus-associated B-cell lymphoma [36]
LINC00657	hsa-miR-378c	Epstein–Barr virus-associated B-cell lymphoma [36]
LINC00657	hsa-miR-378d	Mantle cell lymphoma [37]; Epstein–Barr virus-associated B-cell lymphoma [36]
LINC00657	hsa-miR-378e	Epstein–Barr virus-associated B-cell lymphoma [36]
LINC00657	hsa-miR-378f	Epstein–Barr virus-associated B-cell lymphoma [36]
LINC00657	hsa-miR-378h	Epstein–Barr virus-associated B-cell lymphoma [36]
LINC00657	hsa-miR-378i	Epstein–Barr virus-associated B-cell lymphoma [36]
LINC00657	hsa-miR-421	Diffuse large B cell lymphoma [32]
LINC00657	hsa-miR-422a	Burkitt’s lymphoma [38]
LINC00511	hsa-miR-424-5p	Diffuse large B cell lymphoma [39]
LINC00511	hsa-miR-497-5p	Diffuse large B cell lymphoma [24]
LINC00657	hsa-miR-519d-3p	Diffuse large B cell lymphoma [32]
LINC00657	hsa-miR-520a-3p	Hodgkin’s Lymphoma [30]
LINC00657	hsa-miR-520c-3p	Diffuse large B cell lymphoma [40]
LINC00657	hsa-miR-520d-3p	Diffuse large B cell lymphoma [41]
LINC00657	hsa-miR-92a-3p	Diffuse large B cell lymphoma [42]; Non-Hodgkin’s B cell lymphoma [22]
LINC00657	hsa-miR-92b-3p	Mantle cell lymphoma [22]
**MiRNAs Previously Associated to pSjS**		
**lncRNA**	**miRNA target**	**References**
LINC00657	hsa-miR-106a-5p	[43]
LINC00657	hsa-miR-155-5p	[44]
LINC00511	hsa-miR-15a-5p	[43]
LINC00511	hsa-miR-16-5p	[43]
LINC00657	hsa-miR-17-5p	[43]
LINC00511	hsa-miR-183-5p	[45]
LINC00511	hsa-miR-195-5p	[46]
LINC00657	hsa-miR-20a-5p	[43]
LINC00657	hsa-miR-20b-5p	[43]
LINC00657	hsa-miR-26a-5p	[43]
LINC00657	hsa-miR-30b-5p	[46]
LINC00657	hsa-miR-30c-5p	[46]
LINC00657	hsa-miR-32-5p	[46]
CTD-2020K17.1	hsa-miR-34a-5p	[43]
LINC00657	hsa-miR-378a-3p	[43,46]

## Supplementary Materials

The following are available online at https://www.mdpi.com/2077-0383/8/9/1349/s1, Table S1: Genes modulated in pSjS patients versus healthy subjects, Table S2: LncRNAs modulated in pSjS patients versus healthy subjects, Table S3: Genes modulated in pSjS patients that are targeted by the selected lncRNAs, Table S4: Genes modulated in pSjS patients that are included in the 7 Modules, Table S5: Module-associated modulated genes that are targeted by the selected long non-coding RNAs, Table S6: MiRNAs targets of the selected lncRNAs that regulated genes included in the seven modules, Table S7: Signaling pathways enriched in modulated genes targeted by the selected lncRNAs, Table S8: Signaling pathways enriched in modulated genes included in modules, Figure S1: Validation by RT-PCR. (A) Expression of the three selected lncRNAs in pSjS patients and healthy controls analysed in the study. (B) Expression of selected miRNAs targeted by the three selected lncRNAs in pSjS patients and healthy controls analysed in the study. (C) Expression of selected genes modulated in pSjS samples and healthy controls analysed in the study. Bars indicate SD; *p*-values versus healthy controls <0.001.
