# Supplementary figures and images for: Long Non-Coding RNAs Modulate Sjögren’s Syndrome Associated Gene Expression and Are Involved in the Pathogenesis of the Disease
Source: J Clin Med. 2019 Sep 1;8(9):1349. doi: 10.3390/jcm8091349 (PMC6780488; doi:10.3390/jcm8091349)

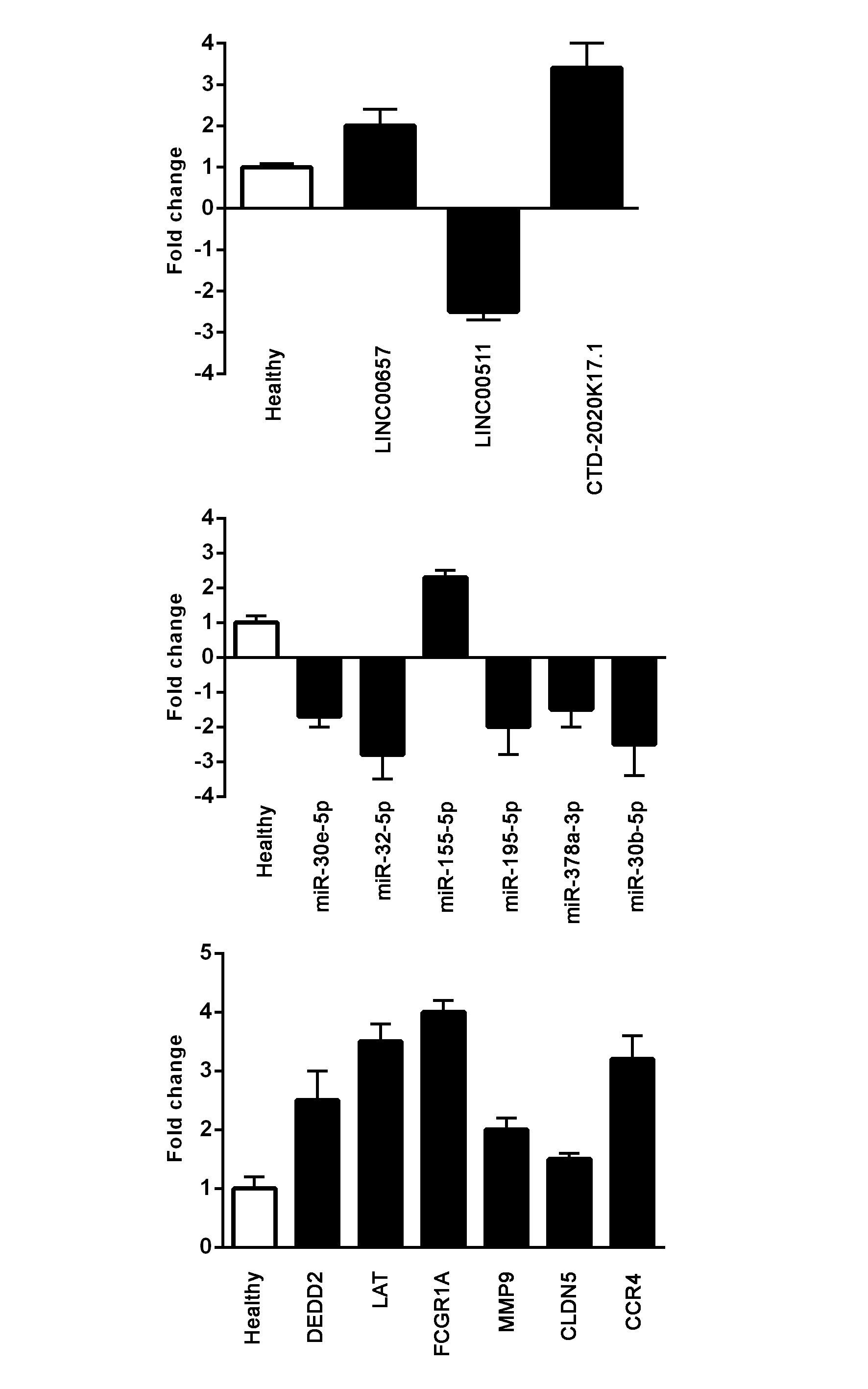

Supplement: Supplementary file 1 [file jcm-08-01349-s001.zip › Supplementary figure 1 pSS.def.tif]
